# Schistosoma Tegument Proteins in Vaccine and Diagnosis Development: An Update

**DOI:** 10.1155/2012/541268

**Published:** 2012-10-18

**Authors:** Cristina Toscano Fonseca, Gardênia Braz Figueiredo Carvalho, Clarice Carvalho Alves, Tatiane Teixeira de Melo

**Affiliations:** ^1^Laboratório de Esquistossomose, Centro de Pesquisas René Rachou, Fundação Oswaldo Cruz, Avenida Augusto de Lima 1715, Belo Horizonte,MG 30190-002, Brazil; ^2^Instituto Nacional de Ciências e Tecnologia em Doenças Tropicais (INCT-DT), Avenida Augusto de Lima 1715, Belo Horizonte, MG 30190-002, Brazil

## Abstract

The development of a vaccine against schistosomiasis and also the availability of a more sensitive diagnosis test are important tools to help chemotherapy in controlling disease transmission. Bioinformatics tools, together with the access to parasite genome, published recently, should help generate new knowledge on parasite biology and search for new vaccines or therapeutic targets and antigens to be used in the disease diagnosis. Parasite surface proteins, especially those expressed in schistosomula tegument, represent interesting targets to be used in vaccine formulations and in the diagnosis of early infections, since the tegument represents the interface between host and parasite and its molecules are responsible for essential functions to parasite survival. In this paper we will present the advances in the development of vaccines and diagnosis tests achieved with the use of the information from schistosome genome focused on parasite tegument as a source for antigens.

## 1. Introduction

Schistosomiasis is still a significant public health problem in tropical countries despite the existence of effective drugs against the parasite [1]. Chemotherapy as a strategy for disease control has proved ineffective in controlling transmission [1] therefore, the development of a vaccine against the disease and also a more sensitive diagnosis test is necessary to assist chemotherapy in control programs [1, 2].

In this context, the recent availability of schistosome genomes information represents an important toll to be used in the discovery of new targets for vaccine and diagnosis. *Schistosoma mansoni* genome, published in 2009 [3] described 11.809 genes while *Schistosoma japonicum* genome [4] has been described to be composed of 13.469 genes. Their assemblies were generated by conventional capillary sequencing resulting in 19.022 scaffolds (*S. mansoni*) and 25.048 scaffolds (*S. japonicum)*. More recently an improved version of the *S. mansoni* genome was published [5], utilizing a combination of traditional Sanger capillary sequencing and deep-coverage Illumina sequencing that refined gene prediction resulting in a reduction in the number of predicted genes from 11.809 to 10.852. Illumina-based technology was also used in *Schistosoma haematobium* genome sequencing, which described 13.073 genes [6].

Simultaneously to genome publication, an important tool to access and analyze parasite genome has been developed, the SchistoDB (http://www.schistodb.net/) database [7]. The SchistoDB enables access to information on the parasite genome even to those researchers not specialized in computer language. The current 3.0 database version provides access to the latest draft of *S. mansoni* genome sequence and annotation and also to *S. japonicum* and *S. haematobium* genome annotation. 

The bioinformatics tools, together with the availability to access parasite genome, should have helped the knowledge of parasite biology and the search for new vaccines, therapeutic targets, and antigens to be used in the disease diagnosis. In this paper we will present the advances in the development of vaccines and diagnostics tests achieved with the use of the information from schistosome genome, focus will be given to the parasite tegument as a source for antigens.

## 2. Host-Parasite Relationship: Role for the Parasite Tegument

Highly adapted to parasitic life, schistosomes can live for years or decades even in a hostile environment as the circulatory system from vertebrate host where the parasite has an intimate contact with circulating elements of the immune system [8]. 

In this successful host-parasite relationship, the host immune system plays an important role in both parasite development and elimination. CD4+ cells, hormones, and cytokines as TNF-*α*, TGF-*β*, and IL-7 produced by the host, seem to assist the parasite development [9–15]. While CD4+ cells, B cells, IFN-*γ*, and TNF-*α* has been described to be involved in parasite elimination in the irradiated cercariae vaccine model [16–18].

Moreover, the highly adapted relationship between schistosomes and the mammalian definitive host also involves the effective mechanisms for evading the immune response that they provoke. In this context, the parasite tegument plays an important role [19, 20]. After penetration, the parasite surface undergoes a profound change that allows parasite adaptation into the host internal microenvironment where the parasite switches from its immune-sensitive to an immune-refractory state [21]. In cercariae, the surface is characterized by a single bilayer membrane covered by a dense glicocalyx. During penetration, the glicocalyx is lost and the membrane transforms into a double bilayer membrane [22]. Evading mechanisms as antigenic mimicry, membrane turnover, production of immunomodulatory molecules and modulation of surface antigens expression also takes place in the parasite surface and contributes to schistosome survival [23, 24].

Trying to eliminate the parasite, host immune system targets the antigens in parasite surface. Studies in mice have shown that the developmental stage most susceptible to the host immune system attack is the schistosomula stage. Very early after infection, schistosomula are susceptible to cellular and humoral immunity, however, in the course of parasite development the susceptibility is rapidly lost [25, 26]. The resistance to host immune response acquired by parasites can be in part explained by surface changes independently of host antigens adsorption [27–29]. In addition, El Ridi and colleagues [30], demonstrated that lung-stage schistosomulum protect themselves from the host immune system by confining antigenic molecules in lipid-rich sites of surface membrane. In contrast, McLaren, in 1989 [31], demonstrated that both skin and lung schistosomula phases are targets of the immune system in the radiation-attenuated vaccine model which trigger an inflammatory reaction around the larvae inhibiting their migration.

Since schistosomula is the major target of the host immune system attack and its tegument represents the interface between parasite and host, also performing vital functions that ensure parasite survival [32], the study of its structure and how it interacts with the host immune system can provide important information about disease control, especially to those related to the search for new drugs and vaccine development. We have recently demonstrated that the schistosomula tegument from *S. mansoni* (Smteg) is recognized by TLR4 in dendritic cells (DC) leading to DC activation and production of proinflammatory cytokines as IL-12 and TNF-*α* [33]. In contrast to this inflammatory profile, Smteg also induce IL-10 production by DC in a TLR (Toll like receptors) 2, 3, 4, and 9 independent manner (unpublished data) once again demonstrating that schistosomula tegument can both activate or modulate host immune system.

## 3. The Tegument as Antigen Source for Vaccine Development

Most of the studies that aimed to identify membrane proteins in parasite tegument were performed in adult worms [34–36]. Although schistosomula is the major target for host immunity, its tegument proteins have still not been characterized, mainly due to the difficulty in obtaining sufficient quantities of material for such protein studies [37]. Indeed protective antigens are found in *S. mansoni* schistosomula tegument (Smteg) since mice immunization with Smteg formulated with Freunds' adjuvant [38] or Alum + CPG-ODN (unpublished data) is able to reduce significantly worm burden and egg elimination with the feces. The characterization of these protective antigens is being performed using immune-proteomics analysis and genome databases to identify candidates to be used in a vaccine formulation against schistosomiasis. Other “omics” technologies are also being used to identify schistosoma proteins, mainly those expressed in schistosomula. In this context, two studies, using cDNA microarrays technologies assessed the most relevant transcriptional changes in the schistosomula development phase. These studies demonstrated that tetraspanin, Sm22.6, Sm29, Sm200 and phosphadiesterase are membrane proteins are highly expressed during schistosomula phase [39, 40]. Furthermore, the studies that used gene silencing through RNAi technique could clarify the importance of some proteins, such as cathepsins [41, 42] and tetraspanins [43] for parasite development and survival. The same membrane protein was identified in adult worm tegument preparations using Mass spectrometry (MS-)-based proteomics [33, 34] together with genome, transcriptome and genetic maps information [3, 44–46]. Recently a proteomic analysis demonstrated that Sm29 and Sm200 are linked to parasite surface membrane through a GPI-anchor [47] while the most abundant protein in adult worm tegument, among the investigated molecules, are aquaporin, dysferlin, TSP-2, and ATP diphosphohydrolase [48]. Among this expressive catalogue of protein expressed in the schistosome tegument, some of them have been evaluated as vaccine antigen in immunization protocols in mice. The Table 1 summarizes the results observed in these preclinical trials using tegument proteins.

Sm29 was identified by Cardoso and coworkers using *in silico* analysis to identify in *S. mansoni* transcriptome putative expressed proteins localized in the parasite tegument [49]. Sm29 recombinant form induces a Th1 profile in mice associated with a reduction of 51% in worm burden when used in vaccine formulation [50]. The tegumental protein, Sm22.6 and its homologue in *S. japonicum* (Sj22.6), are involved in resistance to reinfection in endemic areas [51, 52]. Immunization of mice with recombinant 22.6 formulated with Freund adjuvant resulted in 34.5% reduction on worm burden [53] while Sm22.6 formulated with alum failed to induce protection against schistosomiasis but induced a regulatory response able to modulate allergic asthma in mice [54, 55].

Tetraspanins (TSP) 1 and 2 were identified in a cDNA library from *S. mansoni* based on their membrane-targeting signal [56]. Immunization of mice with TSP1 recombinant protein resulted in a reduction of 57% in worm burden and reduction in the number of eggs in liver (64%) and intestine (65%), TSP2 recombinant protein was less effective in reducing worm burden (34%) but had similar effects in reducing the number of eggs trapped in the liver (52%) and intestine (69%) [57]. The TSP-2 homologue in *S. japonicum* has also been evaluated in murine immunization however no protection was observed [58].

ECL or Sm200 is a GPI-anchored protein in the *S. mansoni* tegument that has also been associated with praziquantel efficacy, since antibodies against this protein can restore drug efficacy in B cells depleted mice [59, 60]. Murine DNA vaccination with the gene encoding Sm200 elicited 38.1% protection while immunization of mice with enzymatically cleaved GPI-anchored proteins from the *S. mansoni* tegument, in which Sm200 represent the most abundant protein result in 43% reduction in adult worm burden [61, 62]. Sm21.7 was tested as antigen in a recombinant vaccine [63] and DNA vaccine [64]. Immunization of mice with recombinant Sm21.7 resulted in a decrease of 41%–70% in worm burden while DNA vaccination resulted in of 41.5% worm burden reduction [63, 64]. 

The schistosome antioxidant enzymes (Cu/Zn superoxide dismutase-SOD, glutathione-S-peroxidase-GPX) are developmentally regulated. The lowest level of gene expression and enzyme-specific activity was found in the larval stages while the highest level of gene expression was observed in adult worms [65–68]. This suggests that antioxidant enzymes are important in immune evasion by adult schistosome parasites [67]. Also RNAi assays demonstrated that knocking down the antioxidants enzymes GPX and GST result in dramatic decreases in sporocysts survival indicating that these enzymes are capable of enhancing parasite survival in an oxidative environment [69]. Mice immunized with the antioxidant enzyme Cu-Zn superoxide dismutase in a DNA vaccine strategy resulted in 44–60% reduction in worm burden [65].

## 4. Antigens to Be Used in Schistosomiasis Diagnostic Test

Currently, all available techniques for the diagnosis of schistosomiasis are characterized by having some limitations, especially when it becomes necessary to detect infection in a large number of patients with low parasite load [70]. One of the initial difficulties in the development of a test for the diagnosis of schistosomiasis is the choice of an appropriate antigen. There are several factors that influence this choice: easily of production, high stability in sample storage, immunogenicity, specificity, and ability to be incorporated to low costs test platforms [71].

In this context, the availability of the complete genome sequences in combination with other technologies such as bioinformatics and proteomics, provides important tolls to seek for an ideal candidate to compose an efficient immunodiagnostic test. With this in mind, our group have recently designed an *in silico* strategy based in the principles of reverse vaccinology, and using a rational criteria to mine candidates in parasite genome to be used in the immunodiagnosis of schistosomiasis [72]. Six antigens were selected based on the evidence of gene expression at different phases of the parasite life cycle in the definitive host, accessibility to host immune system (exposed proteins), low similarity with human and other helminthic proteins, and presence of predicted B cells epitopes (Table 2) [72]. Although our *in silico* analysis led to identification of six candidates, this strategy has not been yet experimentally validated.

Other groups have also used bioinformatics analysis to select target sequence from *S. japonicum* genome to be used for the detection of parasite DNA in blood samples. A 230-bp sequence from the highly repetitive retrotransposon SjR2 was identified and it was demonstrated that PCR test to detect SjR2 is highly sensitive and specific for detection *S. japonicum* infection in the sera of infected rabbits and patients [73]. More recently the same group performed a comparative study to determine the best target to be used in a molecular diagnosis test for schistosomiasis japonicum in 29 retrotransposons identified by bioinformatics analysis. A 303-bp sequence had the highest sensitivity and specificity for the detection of *S. japonicum* DNA in serum samples [74]. 

Proteomics analysis has also been used in the identification of candidates to the immunodiagnosis of schistosomiasis. Western Blot with sera from *S. japonicum* infected rabbit in a two-dimensional gel loaded with adult worm preparation identified 10 spots that were demonstrated by LC/MS-MS to correspond to four different proteins: SjLAP (Leucine aminopeptidases), SjFBPA (fructose-1,6-bisphosphate aldolase), SjGST (Glutathione-S-transferase) and SJ22.6 [75]. Recombinant SjLAP and SjFBPA were tested in ELISA assay and presented high efficacy for the diagnosis of *S. japonicum* infection, with 96.7% specificity for both proteins and 98.1% or 87.8% sensitivity to detect acute and chronically infected individuals, respectively, when SjLAP was used as antigen or a sensitivity of 100% (acute) and 84.7% (chronic infection) when SjFBPA was used as antigen [75]. 

## 5. Other Membrane Proteins Candidates to Be Used in Vaccine Formulation and Diagnosis Tests

Aquaporins are small integral membrane proteins involved in the selective transportation of water and other solutes through plasma membranes of mammals, plants and lower organisms [76]. This protein was described to be abundant in schistosome tegument and due to its physiological function and abundance represent an interesting target to vaccines and diagnosis tests [48]. Characterization of the *S. japonicum* aquaporin-3 using bioinformatics tools demonstrated that this 32.9 kDa transmembrane protein has predicted B cells epitopes with the most likely epitopes present in the N-terminal portion of the protein, located outside the membrane [77]. Other abundant protein in schistosoma tegument is dysferlin, based on analogy with homologues from other organisms, this protein seems to be involved in membrane repair and/or vesicle fusion in tegument surface [34].

ATP-diphosphohydrolases are enzymes involved in ADP and ATP hydrolysis that has been related to host immune system evasion, since this enzyme could hydrolyze the ATP produced in response to parasite induced stress in the endothelio thus modulating the DAMP (danger associated molecular pattern)-mediated inflammatory signaling [78, 79]. In schistosomes two different proteins have been described SmATPDase 1 and SmATPDase2 with approximately 63 and 55 kDa [80, 81]. SmATPDase 1 is located in the border of the tegument while SmATPDase2 is located in internal structure of the tegument syncytium and can be secreted [81]. The immunogenicity of the synthetic peptide (r175–190) from SmATPDase2 has been demonstrated in Balb-c mice, however the protection induced by this epitope has not been evaluated [82].

**Figure 1:**
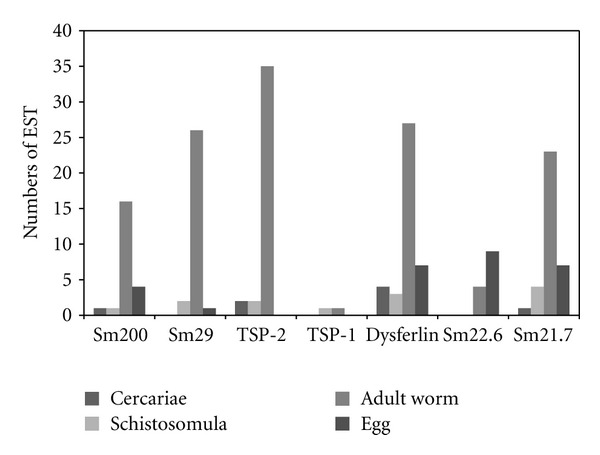
Predicted expression of schistosome tegument proteins in the different parasite life stage in the definitive host. schistosome tegument protein identified by proteomics analysis of the adult worm tegument was analyzed in SchistoDB database (http://www.schistodb.net/). Bars represent the numbers of EST in each parasite life stage whose annotation correspond to Sm200, Sm29, TSP-2, TSP-1, Dysferlin, Sm22.6, or Sm21.7.

Although most tegument protein listed in this paper has been identified in adult worm tegument, an *in silico* analysis performed in SchistoDB (http://www.schistodb.net/) demonstrates that some of them are also expressed in the schistosomula stage as demonstrated in Figure 1 reinforcing their potential to be used in a vaccine formulation or in the early diagnosis of schistosome infection.

## 6. Conclusion

So far the genome, transcriptome, and proteome information provided many targets to be tested in schistosomiasis vaccine and diagnosis and also new knowledge about schistosome biology. However approximately 40% of the schistosome genome is composed of hypothetical proteins with unknown function that represents interesting targets to be tested and characterized. An increase in the knowledge about parasite biology, pathogenesis, and host-parasite relationship can be expected for the next years.

## Figures and Tables

**Table 1:** Schistosome tegument protein evaluated as vaccine candidates in preclinical studies.

Protein	Vaccine type	Protection level	Egg reduction	Bioinformatic tool used in antigen selection	References
Sm 21.7	Recombinant protein	41%–70%	ND	ND	[63]
Sm 21.7	DNA vaccine	41.5%	62% (liver) 67% (intestine)	ND	[64]
Cu/Zn superoxide dismutase	DNA vaccine	44%–60%	ND	ND	[65]
Sm TSP2	Recombinant protein	57%	64% (liver) 65% (feces)	BLAST	[57, 83]
Sm29	Recombinant protein	51%	60% (intestine)	InterProScan, SignalIP 3.0, Signal IP Neural, NetNGlyc 1.0, BLAST, WolfpSORT, SOSUI, Compute pI/Mw tool,	[49, 50]
ECL (200 kDa protein)	DNA vaccine	38.1%	ND	ND	[61]
Sm 22.6	Recombinant protein	34.5%	ND	BLAST	[53]
Sm TSP 1	Recombinant protein	34%	52% (liver) 69% (intestine)	BLAST	[57, 83]

ND: not determined.

**Table 2:** *Schistosoma mansoni* protein selected by genome mining to be used in serological diagnosis for schistosomiasis.

Protein	SchistoDB number	Annotation	Number of amino acid	Base pairs	Predicted molecular weight	Predicted isoelectric point	Predicted location
Sm200	Smp_017730	200-kDa GPI-anchored surface glycoprotein	1656	4971	186,5 kDa	4.97	Tegument surface membranes
Sm12.8	Smp_034420.1	Expressed protein	117	354	12,8 kDa	6.88	Extracellular
Sm43.5	Smp_042910	Expressed protein	382	1149	43,5 kDa	8.43	Extracellular
Sm127.9	Smp_171300	Hypothetical protein	1143	3432	127,9 kDa	6.63	Extracellular
Sm18.9	Smp_184440	Cytochrome oxidase subunit, putative	171	516	18,9 kDa	9.30	Extracellular
Sm16.5	Smp_184550	Cytochrome oxidase subunit, putative	146	441	16,5 kDa	9.14	Extracellular

Adapted of Carvalho et al., 2011 [72].

## References

[1] Bergquist NR, Leonardo LR, Mitchell GF (2005). Vaccine-linked chemotherapy: can schistosomiasis control benefit from an integrated approach?. *Trends in Parasitology*.

[2] Berhe N, Medhin G, Erko B (2004). Variations in helminth faecal egg counts in Kato-Katz thick smears and their implications in assessing infection status with *Schistosoma mansoni*. *Acta Tropica*.

[3] Berriman M, Haas BJ, Loverde PT (2009). The genome of the blood fluke *Schistosoma mansoni*. *Nature*.

[4] Zhou Y, Zheng H, Chen Y (2009). The *Schistosoma japonicum* genome reveals features of host-parasite interplay. *Nature*.

[5] Protasio AV, Tsai IJ, Babbage A (2012). A systematically improved high quality genome and transcriptome of the human blood fluke Schistosoma mansoni. *PLoS Neglected Tropical Diseases*.

[6] Young ND, Jex AR, Li B (2012). Whole-genome sequence of *Schistosoma haematobium*. *Nature Genetics*.

[7] Zerlotini A, Heiges M, Wang H (2009). SchistoDB: a *Schistosoma mansoni* genome resource. *Nucleic Acids Research*.

[8] Harris ARC, Russell RJ, Charters AD (1984). A review of schistosomiasis in immigrants in Western Australia, demonstrating the unusual longevity of *Schistosoma mansoni*. *Transactions of the Royal Society of Tropical Medicine and Hygiene*.

[9] Davies SJ, Grogan JL, Blank RB, Lim KC, Locksley RM, McKerrow JH (2001). Modulation of blood fluke development in the liver by hepatic CD4^+^ lymphocytes. *Science*.

[10] De Mendonça RL, Escrivá H, Bouton D, Laudet V, Pierce RJ (2000). Hormones and nuclear receptors in schistosome development. *Parasitology Today*.

[11] Saule P, Adriaenssens E, Delacre M (2002). Early variations of host thyroxine and interleukin-7 favor *Schistosoma mansoni* development. *Journal of Parasitology*.

[12] Amiri P, Locksley RM, Parslow TG (1992). Tumour necrosis factor *α* restores granulomas and induces parasite egg-laying in schistosome-infected SCID mice. *Nature*.

[13] LoVerde PT, Osman A, Hinck A (2007). *Schistosoma mansoni*: TGF-*β* signaling pathways. *Experimental Parasitology*.

[14] Wolowczuk I, Nutten S, Roye O (1999). Infection of mice lacking interleukin-7 (IL-7) reveals an unexpected role for IL-7 in the development of the parasite *Schistosoma mansoni*. *Infection and Immunity*.

[15] Blank RB, Lamb EW, Tocheva AS (2006). The common *γ* chain cytokines interleukin (IL)-2 and IL-7 indirectly modulate blood fluke development via effects on CD4^+^ T cells. *Journal of Infectious Diseases*.

[16] Vignali DAA, Crocker P, Bickle QD, Cobbold S, Waldmann H, Taylor MG (1989). A role for CD4^+^ but not CD8^+^ T cells in immunity to *Schistosoma mansoni* induced by 20 krad-irradiated and Ro 11-3128-terminated infections. *Immunology*.

[17] Jankovic D, Wynn TA, Kullberg MC (1999). Optimal vaccination against *Schistosoma mansoni* requires the induction of both B cell- and IFN-*γ*-dependent effector mechanisms. *Journal of Immunology*.

[18] Street M, Coulson PS, Sadler C (1999). TNF is essential for the cell-mediated protective immunity induced by the radiation-attenuated schistosome vaccine. *Journal of Immunology*.

[19] Abath FGC, Werkhauser RC (1996). The tegument of *Schistosoma mansoni*: functional and immunological features. *Parasite Immunology*.

[20] Han ZG, Brindley PJ, Wang SY, Zhu C (2009). Schistosoma genomics: new perspectives on schistosome biology and host-parasite interaction. *Annual Review of Genomics and Human Genetics*.

[21] Jones MK, Gobert GN, Zhang L, Sunderland P, McManus DP (2004). The cytoskeleton and motor proteins of human schistosomes and their roles in surface maintenance and host-parasite interactions. *BioEssays*.

[22] Hockley DJ, McLaren DJ (1973). *Schistosoma mansoni*: changes in the outer membrane of the tegument during development from cercaria to adult worm. *International Journal for Parasitology*.

[23] Salzet M, Capron A, Stefano GB (2000). Molecular crosstalk in host-parasite relationships: Schistosome- and leech-host interactions. *Parasitology Today*.

[24] Damian RT (1987). Molecular mimicry revisited. *Parasitology Today*.

[25] Smithers SR, McLaren DJ, Rahalho-Pinto FJ (1977). Immunity to schistosomes: the target. *American Journal of Tropical Medicine and Hygiene*.

[26] Santoro F, Lachmann PJ, Capron A, Capron M (1979). Activation of complement by *Schistosoma mansoni* schistosomula: killing of parasites by the alternative pathway and requirement of IgG for classical pathway activation. *Journal of Immunology*.

[27] Dean DA (1977). Decreased binding of cytotoxic antibody by developing *Schistosoma mansoni*. Evidence for a surface change independent of host antigen adsorption and membrane turnover. *Journal of Parasitology*.

[28] Dessein A, Samuelson JC, Butterworth AE (1981). Immune evasion by *Schistosoma mansoni*: loss of susceptibility to antibody or complement-dependent eosinophil attack by schistosomula cultured in medium free of macromolecules. *Parasitology*.

[29] Racoosin EL, Davies SJ, Pearce EJ (1999). Caveolae-like structures in the surface membrane of *Schistosoma mansoni*. *Molecular and Biochemical Parasitology*.

[30] El Ridi R, Mohamed SH, Tallima H (2003). Incubation of *Schistosoma mansoni* lung-stage schistosomula in corn oil exposes their surface membrane antigenic specificities. *Journal of Parasitology*.

[31] McLaren DJ (1989). Will the real target of immunity to schistosomiasis please stand up. *Parasitology Today*.

[32] Gobert GN, Chai M, McManus DP (2007). Biology of the schistosome lung-stage schistosomulum. *Parasitology*.

[33] Durães FV, Carvalho NB, Melo TT, Oliveira SC, Fonseca CT (2009). IL-12 and TNF-*α* production by dendritic cells stimulated with *Schistosoma mansoni* schistosomula tegument is TLR4- and MyD88-dependent. *Immunology Letters*.

[34] Van Balkom BWM, Van Gestel RA, Brouwers JFHM (2005). Mass spectrometric analysis of the *Schistosoma mansoni* tegumental sub-proteome. *Journal of Proteome Research*.

[35] Braschi S, Borges WC, Wilson RA (2006). Proteomic analysis of the shistosome tegument and its surface membranes. *Memorias do Instituto Oswaldo Cruz*.

[36] Braschi S, Wilson RA (2006). Proteins exposed at the adult schistosome surface revealed by biotinylation. *Molecular and Cellular Proteomics*.

[37] Loukas A, Gaze S, Mulvenna JP (2011). Vaccinomics for the major blood feeding helminths of humans. *OMICS A Journal of Integrative Biology*.

[38] Teixeira De Melo T, Michel De Araujo J, Do Valle Durães F (2010). Immunization with newly transformed *Schistosoma mansoni* schistosomula tegument elicits tegument damage, reduction in egg and parasite burden. *Parasite Immunology*.

[39] Dillon GP, Feltwell T, Skelton JP (2006). Microarray analysis identifies genes preferentially expressed in the lung schistosomulum of *Schistosoma mansoni*. *International Journal for Parasitology*.

[40] Gobert GN, Tran MH, Moertel L (2010). Transcriptional changes in *Schistosoma mansoni* during early schistosomula development and in the presence of erythrocytes. *PLoS Neglected Tropical Diseases*.

[41] Correnti JM, Brindley PJ, Pearce EJ (2005). Long-term suppression of cathepsin B levels by RNA interference retards schistosome growth. *Molecular and Biochemical Parasitology*.

[42] Morales ME, Rinaldi G, Gobert GN, Kines KJ, Tort JF, Brindley PJ (2008). RNA interference of *Schistosoma mansoni* cathepsin D, the apical enzyme of the hemoglobin proteolysis cascade. *Molecular and Biochemical Parasitology*.

[43] Tran MH, Freitas TC, Cooper L (2010). Suppression of mRNAs encoding tegument tetraspanins from *Schistosoma mansoni* results in impaired tegument turnover. *PLoS pathogens*.

[44] Liu F, Cui SJ, Hu W, Feng Z, Wang ZQ, Han ZG (2009). Excretory/secretory proteome of the adult developmental stage of human blood fluke, *Schistosoma japonicum*. *Molecular and Cellular Proteomics*.

[45] Verjovski-Almeida S, DeMarco R, Martins EAL (2003). Transcriptome analysis of the acoelomate human parasite *Schistosoma mansoni*. *Nature Genetics*.

[46] Criscione CD, Valentim CLL, Hirai H, LoVerde PT, Anderson TJC (2009). Genomic linkage map of the human blood fluke *Schistosoma mansoni*. *Genome Biology*.

[47] Castro-Borges W, Dowle A, Curwen RS, Thomas-Oates J, Wilson RA (2011). Enzymatic shaving of the tegument surface of live schistosomes for proteomic analysis: a rational approach to select vaccine candidates. *PLoS Neglected Tropical Diseases*.

[48] Castro-Borges W, Simpson DM, Dowle A (2011). Abundance of tegument surface proteins in the human blood fluke *Schistosoma mansoni* determined by QconCAT proteomics. *Journal of Proteomics*.

[49] Cardoso FC, Pinho JMR, Azevedo V, Oliveira SC (2006). Identification of a new *Schistosoma mansoni* membrane-bound protein through bioinformatic analysis. *Genetics and Molecular Research*.

[50] Cardoso FC, Macedo GC, Gava E (2008). *Schistosoma mansoni* tegument protein Sm29 is able to induce a Th1-type of immune response and protection against parasite infection. *PLoS Neglected Tropical Diseases*.

[51] Dunne DW, Webster M, Smith P (1997). The isolation of a 22 kDa band after SDS-PAGE of *Schistosoma mansoni* adult worms and its use to demonstrate the IgE responses against the antigen(s) it contains are associated with human resistance to reinfection. *Parasite Immunology*.

[52] Santiago ML, Hafalla JCR, Kurtis JD (1998). Identification of the *Schistosoma japonicum* 22.6-kDa antigen as a major target of the human IgE response: similarity of IgE-binding epitopes to allergen peptides. *International Archives of Allergy and Immunology*.

[53] Pacífico LGG, Fonseca CT, Chiari L, Oliveira SC (2006). Immunization with *Schistosoma mansoni* 22.6 kDa antigen induces partial protection against experimental infection in a recombinant protein form but not as DNA vaccine. *Immunobiology*.

[54] Pacífico LGG, Fonseca CT, Barsante MM, Cardoso LS, Araújo MI, Oliveira SC (2006). Aluminum hydroxide associated to *Schistosoma mansoni* 22.6 kDa protein abrogates partial protection against experimental infection but not alter interleukin-10 production. *Memorias do Instituto Oswaldo Cruz*.

[55] Cardoso LS, Oliveira SC, Góes AM (2010). *Schistosoma mansoni* antigens modulate the allergic response in a murine model of ovalbumin-induced airway inflammation. *Clinical and Experimental Immunology*.

[56] Smyth D, McManus DP, Smout MJ, Laha T, Zhang W, Loukas A (2003). Isolation of cDNAS encoding secreted and transmembrane proteins from *Schistosoma mansoni* by a signal sequence trap method. *Infection and Immunity*.

[57] Tran MH, Pearson MS, Bethony JM (2006). Tetraspanins on the surface of *Schistosoma mansoni* are protective antigens against schistosomiasis. *Nature Medicine*.

[58] Zhang W, Li J, Duke M (2011). Inconsistent protective efficacy and marked polymorphism limits the value of *Schistosoma japonicum* tetraspanin-2 as a vaccine target. *PLoS Neglected Tropical Diseases*.

[59] Sauma SY, Strand M (1990). Identification and characterization of glycosylphosphatidylinositol-linked *Schistosoma mansoni* adult worm immunogens. *Molecular and Biochemical Parasitology*.

[60] Brindley PJ, Strand M, Norden AP, Sher A (1989). Role of host antibody in the chemotherapeutic action of praziquantel against *Schistosoma mansoni*: identification of target antigens. *Molecular and Biochemical Parasitology*.

[61] Nascimento EJM, Amorim RV, Cavalcanti A (2007). Assessment of a DNA vaccine encoding an anchored- glycosylphosphatidylinositol tegumental antigen complexed to protamine sulphate on immunoprotection against murine schistosomiasis. *Memorias do Instituto Oswaldo Cruz*.

[62] Martins VP, Pinheiro CS, Figueiredo BCP (2012). Vaccination with enzymatically cleaved GPI-anchored proteins from schistosoma mansoni induces protection against challenge infection. *Clinical and Developmental Immunology*.

[63] Ahmed HM, Romeih MH (2001). Protection against *Schistosoma mansoni* infection with recombinant schistosomula 21.7 kDa protein. *Arab Journal of Biotechnology*.

[64] Ahmed HM, Romeih MH, Abou-Shousha TS (2006). DNA immunization with the gene encoding Sm21.7 protects mice against *S. mansoni* infections. *American Journal of Science*.

[65] Shalaby KA, Yin L, Thakur A, Christen L, Niles EG, LoVerde PT (2003). Protection against *Schistosoma mansoni* utilizing DNA vaccination with genes encoding Cu/Zn cytosolic superoxide dismutase, signal peptide-containing superoxide dismutase and glutathione peroxidase enzymes. *Vaccine*.

[66] Hong Z, Kosman DJ, Thakur A, Rekosh D, LoVerde PT (1992). Identification and purification of a second form of Cu/Zn superoxide dismutase from *Schistosoma mansoni*. *Infection and Immunity*.

[67] Mei H, LoVerde PT (1997). *Schistosoma mansoni*: the developmental regulation and immunolocalization of antioxidant enzymes. *Experimental Parasitology*.

[68] Mei H, Thakur A, Schwartz J, Lo Verde PT (1996). Expression and characterization of glutathione peroxidase activity in the human blood fluke *Schistosoma mansoni*. *Infection and Immunity*.

[69] Mourão MDM, Dinguirard N, Franco GR, Yoshino TP (2009). Role of the endogenous antioxidant system in the protection of *Schistosoma mansoni* primary sporocysts against exogenous oxidative stress. *PLoS Neglected Tropical Diseases*.

[70] Hamilton JV, Klinkert M, Doenhoff MJ (1998). Diagnosis of schistosomiasis: antibody detection, with notes on parasitological and antigen detection methods. *Parasitology*.

[71] Doenhoff MJ, Chiodini PL, Hamilton JV (2004). Specific and sensitive diagnosis of schistosome infection: can it be done with antibodies?. *Trends in Parasitology*.

[72] Carvalho GBF, da Silva-Pereira RA, Pacífico LGG, Fonseca CT (2011). Identification of Schistosoma mansoni candidate antigens for diagnosis of schistosomiasis. *Memorias do Instituto Oswaldo Cruz*.

[73] Xia CM, Rong R, Lu ZX (2009). *Schistosoma japonicum*: a PCR assay for the early detection and evaluation of treatment in a rabbit model. *Experimental Parasitology*.

[74] Guo J-J, Zheng H-J, Xu J, Zhu X-Q, Wang S-Y, Xia C-M (2012). Sensitive and specific target sequences selected from retrotransposons of *Schistosoma japonicum* for the diagnosis of schistosomiasis. *PLoS Neglected Tropical Diseases*.

[75] Zhong ZR, Zhou HB, Li XY (2010). Serological proteome-oriented screening and application of antigens for the diagnosis of *Schistosomiasis japonica*. *Acta Tropica*.

[76] Verkman AS (2002). Physiological importance of aquaporin water channels. *Annals of Medicine*.

[77] Song J, He Q-F (2012). Bioinformatics analysis of the structure and linear B-cell epitopes of aquaporin-3 from *Schistosoma japonicum*. *Asian Pacific Journal of Tropical Medicine*.

[78] Vasconcelos EG, Nascimento PS, Meirelles MNL, Verjovski-Almeida S, Ferreira ST (1993). Characterization and localization of an ATP-diphosphohydrolase on the external surface of the tegument of *Schistosoma mansoni*. *Molecular and Biochemical Parasitology*.

[79] Bhardwaj R, Skelly PJ (2009). Purinergic signaling and immune modulation at the schistosome surface?. *Trends in Parasitology*.

[80] DeMarco R, Kowaltowski AT, Mortara RA, Verjovski-Almeida S (2003). Molecular characterization and immunolocalization of *Schistosoma mansoni* ATP-diphosphohydrolase. *Biochemical and Biophysical Research Communications*.

[81] Levano-Garcia J, Mortara RA, Verjovski-Almeida S, DeMarco R (2007). Characterization of *Schistosoma mansoni* ATPDase2 gene, a novel apyrase family member. *Biochemical and Biophysical Research Communications*.

[82] Mendes RGPR, Gusmão MAN, Maia ACRG (2011). Immunostimulatory property of a synthetic peptide belonging to the soluble ATP diphosphohydrolase isoform (SmATPDase 2) and immunolocalisation of this protein in the schistosoma mansoni egg. *Memorias do Instituto Oswaldo Cruz*.

[83] Hemler ME (2001). Specific tetraspanin functions. *Journal of Cell Biology*.

